# An Article for Every Family

**Published:** 1884-06

**Authors:** 


### AN ARTICLE FOR EVERY FAMILY.

Dr. Jerome Kidders Electro-Medical Apparatus have so long been in use and are so favorably known that they need only a mention from us, and no recommendation to the profession. A careful reading of the book of instructions which accompanies the Family Faradic Battery will enable anyone to operate effectually for the cure of various diseases. As has been said, "Electricity is life"—it is Nature's own remedy, and does not leave the system filled with poison, as is the case in taking medicine. The Jerome Kidder Manufacturing Co. of this city have received the "Medal of Superiority" from the American Institute for the Fall of 1883, over three competitors, for their superior Electro-Medical Apparatus. This old-established house has no equal for the excellence, both of design and manufacture, which renders their machines a standard of quality all over the country. This Company still remains at 820 Broadway, and will gladly furnish any with circulars, giving a description of their several instruments which are so potent in the eradication of disease and rstoration of health.

